# Ultrabroadband terahertz-band communications with self-healing bessel beams

**DOI:** 10.1038/s44172-023-00118-8

**Published:** 2023-10-06

**Authors:** Innem V.A.K. Reddy, Duschia Bodet, Arjun Singh, Vitaly Petrov, Carlo Liberale, Josep M. Jornet

**Affiliations:** 1 Department of Electrical Engineering, University at Buffalo SUNY, Buffalo, NY USA; 2 Biological and Environmental Science and Engineering Division, King Abdullah University of Science and Technology, Thuwal, Saudi Arabia; 3 Department of Electrical and Computer Engineering, Institute for the Wireless Internet of Things, Northeastern University, Boston, MA USA; 4 Department of Engineering, SUNY Polytechnic Institute, Utica, NY USA; 5 Computer, Electrical and Mathematical Sciences and Engineering Division, King Abdullah University of Science and Technology, Thuwal, Saudi Arabia

**Keywords:** Electrical and electronic engineering, Information theory and computation

## Abstract

The large available bandwidth at sub-terahertz and terahertz frequencies has the potential to enable very high data rates for wireless communications. Moreover, given the large electrical size of terahertz antenna apertures, many future terahertz communication systems will likely operate in the near field. However, due to their reliance on highly directional beams, terahertz systems are susceptible to blockage. Here, we propose using Bessel beams to overcome issues caused by blockage due to their diffraction-free nature and self-healing properties in the near field. We compare the performance of information-bearing Bessel beams and Gaussian beams with and without an obstacle. We later discuss the use of reconfigurable intelligent surfaces to construct terahertz Bessel beams. Finally, we propose a metric to quantify the quality of imperfectly generated terahertz Bessel beams and explore their ability to self-heal. The results demonstrate that Bessel beams are an attractive option for near-field terahertz communications, especially when mitigating the effects of partial blockage.

## Abstract (web summary)

Innem Reddy and colleagues experimentally showcase data communications at speeds of up to 40 gigabits per second at terahertz frequencies with Bessel beams and validate their self-healing nature using a physical obstacle. The results suggest Bessel beams could be used for THz near-field communications, especially when mitigating the effects of blockages.

## Introduction

In the last decade, there has been a tremendous increase in the research focused on terahertz (THz) systems. It is an emerging field with vast opportunities in imaging, sensing, and wireless communications. The THz domain is defined to have frequencies ranging from 100 GHz to 10 THz, with frequencies below 300 GHz classified as sub-THz. The availability of large bandwidths makes this regime more appealing to realize high-data-rate communications, which is why next-generation communications will likely operate in this domain^1^. However, this benefit comes with a price. The high path loss experienced by terahertz frequencies often necessitates a line-of-sight propagation path, which can be easily blocked by most everyday objects. Additionally, the water absorption coefficient is high in this regime, and since most living organisms are composed of water, they can absorb a substantial amount of incident radiation. Thus, if something or someone is obstructing or passing across an active communication link, brief signal disruptions are likely. Even partial blockage can lead to substantial degradation in performance. Intelligent reflecting surfaces (IRSs) can be used to enable non-line-of-sight transmissions^2,3^, but such surfaces are large and require full channel state information to overcome blockage^4^.

Another unique aspect of terahertz communications, different from both microwave and millimeter (mmWave) communications, is that the receiver can often be in the near field of the transmitter’s radiation pattern^5,6^. This phenomenon is due to the small wavelength of terahertz frequencies compared to the large size of the radiating or reflecting device. For example, the Fraunhofer region of a *D* = 10 cm antenna or antenna array at 300 GHz, given by 2*D*^2^/*λ* (where *D* is the aperture of the antenna and *λ* is the wavelength), starts at 20 m. As a result, common far-field beamsteering will not be optimal^7^, but an alternative method of overcoming obstacles, namely near-field wavefront engineering and subsequent beam-shaping, becomes viable. To this end, we propose near-field solutions, specifically Bessel beams (BBs), as a potential solution to mitigate the effects of blockage in a THz communication system.

Durnin first introduced BBs through his seminal papers^8,9^ published in 1987. As time progressed, BBs gained prominence due to their distinctive properties, such as diffraction-free propagation and self-healing nature. These properties are a result of BBs’ unique amplitude and phase profile. Due to their unparalleled attributes, BBs have found applications in several fields^10^ ranging from optics^11^ and biophotonics^12^ to quantum communications^13^. Some notable applications include trapping and manipulating microscopic particles^14^, optical coherence tomography^15^, light-sheet microscopy^16^, etc. There are several ways to generate these BBs: transforming a ring-shaped beam with a lens^17^, using a conical optical element called an axicon^18^, using computer-generated holograms^19^ or metasurfaces^20^, etc. Out of these methods, an axicon is one of the most commonly used tools to transform conventional Gaussian beams (GBs) to BBs due to its simplicity. In general, BBs can be organized into two categories, namely, zeroth-order BBs and higher-order BBs. Zeroth-order BBs contain a high-intensity central spot surrounded by rings, while higher-order BBs contain a dark center with rings around it. In this paper, we primarily focus on the zeroth-order BBs.

BBs in the THz domain are still under preliminary investigation. A proliferation in the availability of THz sources and 3D-printed optics has sped up the usage of THz BBs. Nonetheless, there are only a few works to date that demonstrate the generation and application of THz BBs. In 2003, Lloyd et al.^21^ used a teflon axicon to generate BBs and study superluminal effects with THz time-domain spectroscopy (TDS). This work is the first-ever THz BBs application, to the best of our knowledge. Later, in 2005, Trappe et al.^22^ used a high-density polyethylene axicon to generate THz BBs. Subsequently, several articles illustrated THz BBs using axicons made of polytetrafluoroethylene^23–26^, high-density polyethylene^27,28^, polylactic acid^29^, silicon dioxide^30^, etc. Some works presented THz BBs without axicons but instead by utilizing parallel-plate waveguides^31,32^ and metasurfaces^33^. An alternative way of generating THz BBs is through the utilization of antenna arrays, wherein the required conical phase profile is mapped in the form of a phase codebook across the array elements^34^. In this direction, programmable metasurfaces, composed of sub-wavelength radiating elements packed in a dense configuration are now being explored to constitute a near-continuous reconfigurable radiating sheet, thus acquiring the name of reconfigurable intelligent surfaces (RIS). Through such devices, it should be possible to dynamically control and modify the reflection/transmission of an incident wave or a wave generated by the metasurface itself. In the THz regime, both transmitting metasurfaces and reflecting metasurfaces have been explored. In transmission, the RIS dynamically adapts the incident waveform as it passes through the metasurface^35,36^, whereas in reflection, similar to IRSs, the metasurface implements a response on an incident wave and then reflects the configured signal. Metasurface-based IRSs have been explored to create virtual line-of-sight (LOS) paths in the presence of blockage for mobile users or environments^3,37^. The primary motivation for employing RISs is that they can overcome high propagation losses of single antenna systems by utilizing beamforming methods^38,39^. Also, RISs provide the opportunity for dynamic beamshaping with tailored beam properties, by carefully controlling the phase of each radiating element. Within the past decade, many array architectures have been designed for THz communication systems^40–44^. These designs only discuss the possibility of a practical communication system and do not include the generation of BBs.

In this paper, we experimentally demonstrate high-data-rate THz BB communications and compare the results with standard Gaussian beam communications. The published articles mentioned above, specifically the single antenna-based BBs generation, are primarily associated with the generation of THz BBs and their applications in imaging. BBs have a more extensive diffraction-free length than Gaussian beams and can substantially improve depth of focus along with an increase in resolution. Prior works focused on this aspect and showed applications such as computed tomography, increased quality in the captured image due to higher resolution, etc. However, there are no published works that use THz BBs for high-data-rate communications, and our work is the first one to illustrate the applications in the field of communications. In this article, we also demonstrate the performance of BBs in overcoming obstacles during communication and perform quantitative analyses to showcase the benefit of THz BBs. We then extend our study to RIS-generated BBs, as such a system can replace single antenna-based communications in the future. We perform this analysis theoretically using MATLAB. In an RIS system, the spacing between the elements plays a critical role and can affect the quality of the generated beam. Our theoretical model can determine the relationship between antenna elements’ physical dimensions in an array, the quality of generated BBs, and its ability to reconstruct after an obstacle.

The work in this paper is organized as follows. In the results section, we first experimentally demonstrate that BBs can carry data and compare the bit error rate with conventional Gaussian beams. Then, we introduce an obstacle and transmit data with and without axicon (i.e., generating BBs and Gaussian beams, respectively) and experimentally compare their performance. Next, we provide a method to numerically assess the quality of RIS-generated BBs and use this assessment to analyze the RIS-generated BBs’ ability to reconstruct. In the discussion section, we provide our insights on the attractive use of THz BBs in the next-generation THz communication systems. We summarize and conclude our work in the conclusion section, and we present any additional information on our process in the Methods section.

## Results

In this paper, we perform two different analyses to demonstrate the validity of THz BBs for near-field communications. First we use a state-of-the-art THz testbed^45,46^, to compare the performance of axicon-generated BBs and standard Gaussian beams for communications with and without a partial blocker. Axicons, however, will likely not be used to generate BBs used for THz communications because axicons must be custom-built for any given scenario; RIS-generated BBs are much more likely. Thus our second contribution is to assess the quality and performance of RIS-generated BBs using a MATLAB simulation.

### Experimental demonstration of sub-THz information-bearing bessel beams as a potential near-field communication solution

We use the TeraNova testbed (described further in Methods and extensively by refs. ^45,46^) to generate modulated signals with bandwidths of up to 20 GHz at a carrier frequency of 130 GHz. The transmitting antenna includes a lens that generates a collimated GB in front of which we can place a 3-D printed axicon to transform the GB into a zeroth-order BB. Given the antenna size of 118 mm, the far field of this antenna begins at around 12 m. Therefore, all experiments for this work are performed in the near field.

**Fig. 1:**
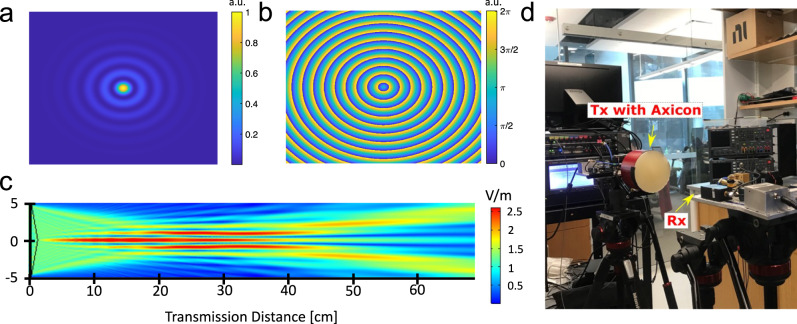
Terahertz Bessel beam (BB) generation. **a** Amplitude profile of a generic BB; **b** Phase profile of the same BB shown in (**a**); **c** A 2D simulation (XZ-profile) of BB generated through the designed axicon; **d** terahertz testbed with an axicon mounted to the transmitter (Tx) while the receiver (Rx) is placed 40 cm away from the Tx.

The generated BB contains a high-intensity center surrounded by rings, as shown in Fig. 1a. The phase profile of this beam is no longer spatially uniform and contains step-like variation radially, as shown in Fig. 1b. During our study, we transmitted modulated signals using BBs and compared the performance of the same signals transmitted using traditional GBs.

#### Improved communication performance for bessel beams vs. Gaussian beams in the near-field line-of-sight scenario

**Fig. 2:**
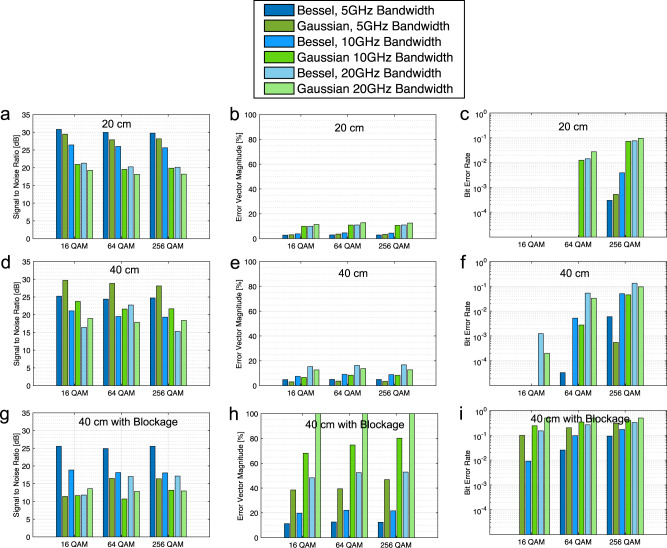
Signal-to-Noise Ratio (SNR), Error Vector Magnitude (EVM), and Bit Error Rate (BER) values from experimental results of various Quadrature Amplitude Modulated (QAM) waveforms. **a**–**c** Results with the receiver placed at 20 cm with no blockage (i.e., within the range of the Bessel beam), **d**–**f** with the receiver placed 40 cm with no blockage, and (**g**–**i**) at 40 cm with blockage placed at 20 cm.

We start with the receiver at 20 cm to keep it within the focal length of the BB. As seen in Fig. 1c, the BB’s focus lasts from around 4 cm to 35 cm. The experimental set-up is shown in Fig. 1d, and the results are shown in the first row of Fig. 2. The results for BBs are shown in blue while the GB results are shown in green with various shades corresponding to different transmitted bandwidths. Thus in the figure, we are primarily comparing adjacent blue (Bessel) and green (Gaussian) bars. From Fig. 2a, we see that the signal-to-noise ratio (SNR) is higher for the BB than it is for the GB in all cases. This gain corresponds to the gain we would anticipate from the focusing of the axicon. Correspondingly, in Fig. 2b the Error Vector Magnitude (EVM) for the Bessel case is lower than the Gaussian case, and the Bit Error Rate (BER) in Fig. 2c follows the same trend. Furthermore, considering a forward error correction limit of 10^−2^, using a BB instead of a GB can enable higher data rates. For example, with 10 GHz of bandwidth and carrying a 256-QAM, the BB is able to keep its BER performance below the limit, while the GB is not. Thus, given the higher focusing power of the axicon, these results verify that BBs provide improved performance in the THz near field when compared with traditional GBs.

**Fig. 3:**
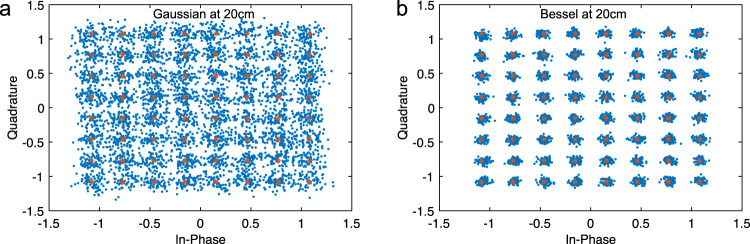
Constellation Diagrams at 20 cm. Received and expected constellation diagrams for 10 GHz Bandwidth 64 Quadrature Amplitude Modulated (64 QAM) signal - the received constellations when using the (**a**) Gaussian beam, and the (**b**) Bessel beam.

We can also observe this improved performance by comparing the received IQ-constellations using a Gaussian beam in Fig. 3a with the received IQ-constellation using a Bessel beam in Fig. 3b. The expected constellation symbols are shown in red, while the demodulated symbols are plotted in blue. For the Bessel Beam, we see the received symbols clustering more tightly around the expected points, corresponding to the lower EVM and leading to fewer bit errors. Meanwhile, the Gaussian beam’s symbols are demodulated less accurately.

#### Demonstration of bessel beams mitigating effects of partial blockage

**Fig. 4:**
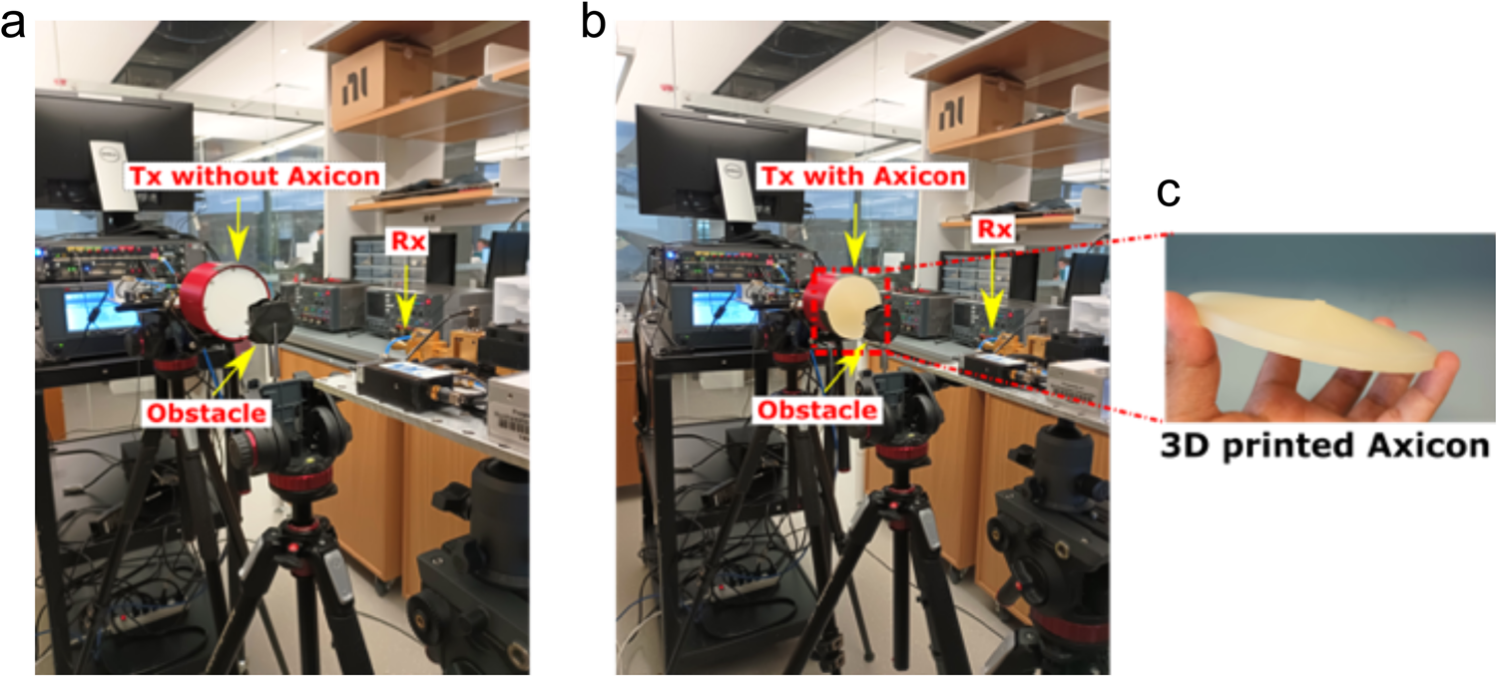
Experimental Setup with Blockage. Experimental setup to illustrate the self-healing capabilities of a Bessel beam (BB);—a cylindrical obstacle containing a wet soaked fabric placed in-between the transmitter (Tx) and the receiver (Rx)—**a** the Tx emits a Gaussian beam; **b** the Tx equipped with an axicon emits a BB; **c** 3D printed axicon mounted on the Tx.

Next, we place the receiver (Rx) 40 cm away from the transmitter (Tx) as shown in Fig. 4. Also considering the focal length of the generated BB, shown in Fig. 1c, we were operating at the tail end of the BB’s focal range. As a result, for these experiments we were operating in the near field of the collimated Gaussian beam and at the edge of the non-diffracting and self-healing region of the Bessel beam.

After observing the unobstructed case, we introduced an obstacle half-way between the Tx and Rx, at 20 cm from the source as shown in Fig. 4a, b. The obstacle was a hollow plastic cylinder with a diameter of 5 cm and a height of 2 cm holding a fabric soaked in water, mimicking a perfectly absorbing medium. The central symmetry axis of the obstacle was manually aligned with Tx–Rx axis and the axicon shown in Fig. 4c.

**Fig. 5:**
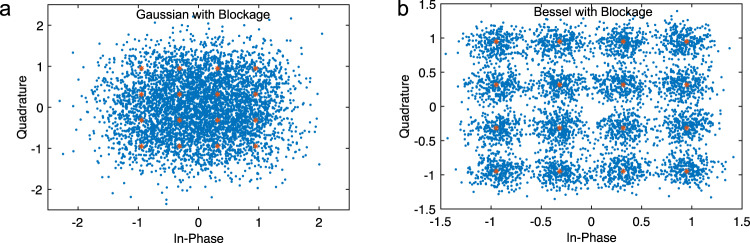
Constellation Diagrams at 40 cm with Blockage. Received and expected constellation diagrams for 5 GHz Bandwidth 16 Quadrature Amplitude Modulated (16 QAM) signal in the presence of blockage—the received constellations when using the (**a**) Gaussian beam, and the (**b**) Bessel beam.

Figure 2d, e and f summarizes the observed performances of both Bessel and Gaussian beams over a 40 cm transmission distance without blockage, while Fig. 2g, h and i, present the results with the blocking element placed 20 cm from the transmitter. Figure 5a, b shows the received constellation diagrams for a 16 QAM signal sent with 5 GHz of bandwidth. Considering these results there are several observations that we would like to highlight:

As shown in Fig. 1c, the Bessel Beam is beginning to diverge close to 40 cm, so we do not observe a gain compared to the Gaussian beam in the unobstructed case. The observed SNR for the Gaussian beams (in green) still outperforms the Bessel (in blue) in this case. Adding the absorbing material as blockage (i.e., comparing Fig. 2d with Fig. 2g), the SNR observed from the Bessel beams drops by only a few dB. Meanwhile, the Gaussian beams’ SNR drops by as much as 14 dB. Furthermore, for the 20 GHz bandwidth signals, the receiver was not able to detect the GB at all (hence the EVM values of 100% and BERs on the order of 0.5 in Figure 2h and i, respectively), but it was still able to detect the BB. This difference clearly demonstrates the Bessel beam’s capability to reconstruct and overcome the obstacle.

The error vector magnitude (EVM), shown in Fig. 2b, e and h of a received waveform is generally worse (i.e., higher) for lower SNR values, which is what we observe here. We also expect, BER to be directly proportional to EVM, which is demonstrated by Fig. 2b, e and h as well. For both the unobstructed and obstructed cases, we also observe other trends we would expect. Namely, as the bandwidth increases, the SNR decreases due to more noise being captured with the signal.

We would also like to point out that despite observing worse power and having a lower SNR than the Gaussian beam in this unobstructed case, the Bessel beam BER performance is comparable to that of the Gaussian for the lower modulations, which indicates that even without obstruction and at the edge of their focal range, Bessel beams are still viable options for THz near-field communications.

Further analysis on transmitting images using BBs and Gaussian beams in the presence of an obstacle is mentioned in Supplementary Note 1.

### Generation of THz Bessel beams with reconfigurable intelligent surfaces

#### Evaluating the purity of RIS-generated bessel beams

**Fig. 6:**
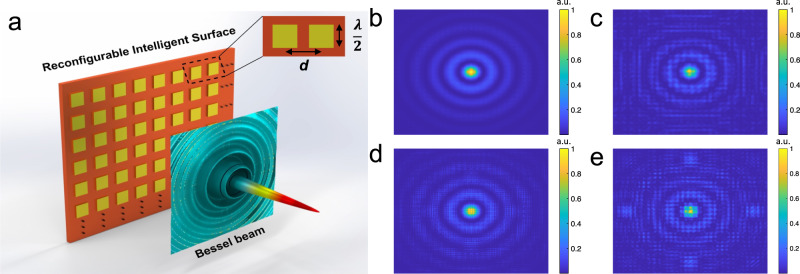
Array-based Bessel beam (BB) generation. **a** A picture representation of antenna array system generating BB. The width and spacing between the antenna elements are mentioned in the inset; **b**–**e** generated BBs while varying the antenna spacing. Here, the spacing, *d*, is 0, *λ*/2, 2*λ*/3, and *λ* respectively, where *λ* is the carrier wavelength.

Here, we perform a theoretical analysis on the generation of BBs with an antenna array, we estimate the quality of the generated BBs, and evaluate their ability to reconstruct after an obstacle. The RIS chosen for simulations could be implemented using an antenna array or a metasurface, wherein the radiating elements as well as the spacing in between them are sub-wavelength. The array consists of patch antennas with half-wavelength dimensions. A pictorial representation of the array generating a BB is shown in Fig. 6a. For the analysis, the array is operated at a design frequency of 130 GHz, with a size of 1 × 1 m with the individual patch width equal to half the wavelength (1.15 mm), as shown in the inset of Fig. 6a. We used a parametric model where we varied the spacing between the elements, *d*, to study the quality of the generated BBs. As we increase the spacing, the effective phase across the RIS becomes more discretized and will eventually affect the quality of the generated BBs. Figure 6b–e showcases the generated BBs when antenna spacing, *d*, is 0, *λ*/2, 2*λ*/3, and *λ*, respectively, where *λ* is the wavelength. As the spacing between the antenna elements increases it leads to a coarser discretization of the applied spatial phase, and the quality of generated BBs dropped substantially.

Although the reduction in quality is evident, there is no well-established approach to quantitatively estimate this degradation. Here, we propose a technique to assess the purity of generated BB. In the RIS configuration, as the antenna elements’ spacing increases, the phase displayed on the array becomes more discrete and may lead to the generation of impure Bessel beams. A simple way to assess the quality of the generated beams is by taking a spatial Fourier transform. While a perfect BB transforms into an annular beam with zero intensity in the center^9^, the imperfect discretization of phase by the RIS introduces low-spatial-frequency components in the Fourier domain. Thus, the Fourier transform of an “impure” BB from the RIS will lead to an annular beam along with a certain non-zero intensity strength in the center. A quantitative measure of the purity of a BB could be obtained by using the formula

1 $$Purity=\left(1-\frac{Pi{x}_{BB}}{Pi{x}_{GB}}\right),$$

where *Pix*_*BB*_ is the central pixel intensity of the Fourier transform of the generated beam from RIS configuration, and *Pix*_*GB*_ is the central pixel intensity of the Fourier transform of the incident beam, that is, a Gaussian beam. When the spacing between antenna elements in the RIS configuration tend to ∞ the incident beam passes through without accumulating any phase, and hence we normalized the *Pix*_*BB*_ value with *Pix*_*GB*_.

**Fig. 7:**
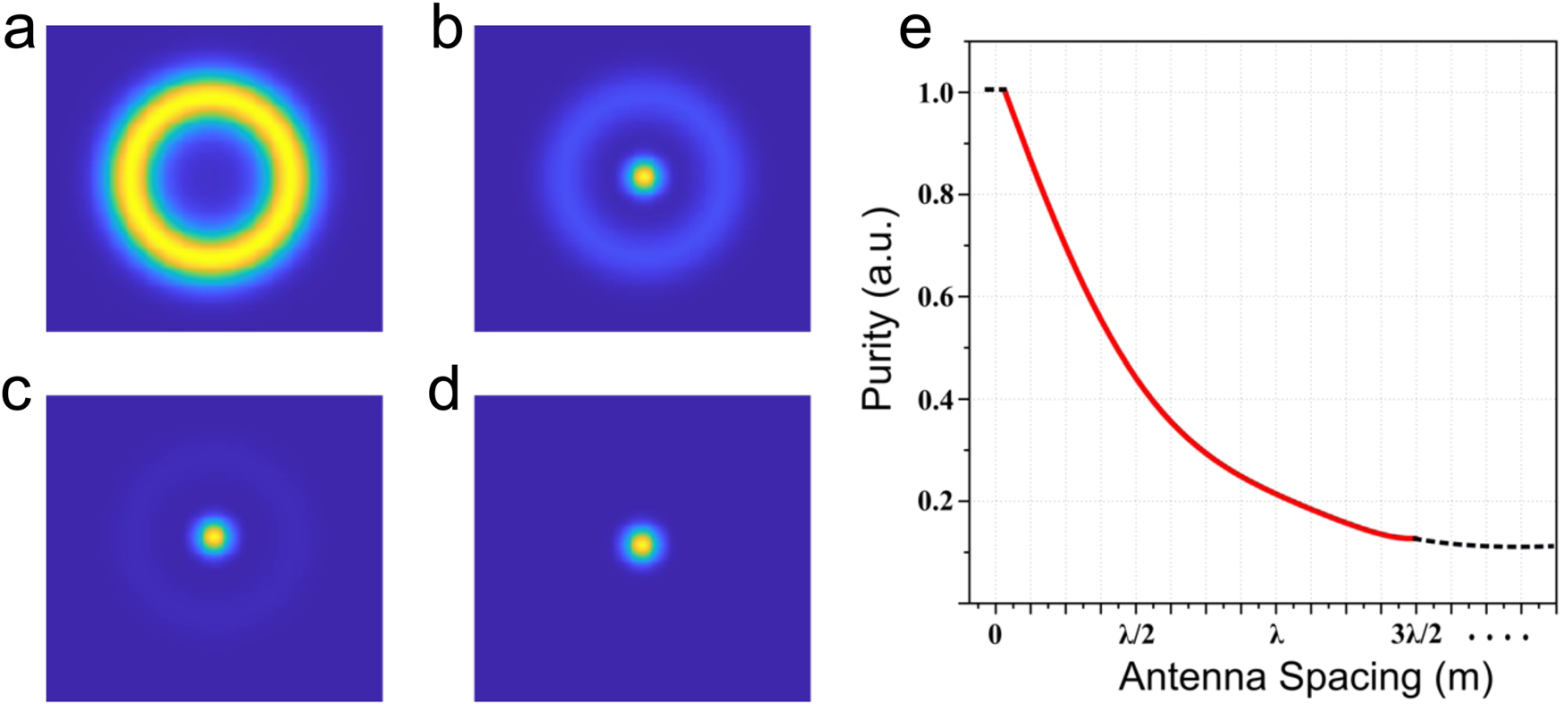
Purity assessment of the generated Bessel beams (BBs). **a**–**d** Fourier transform of the generated BBs when the antenna spacing, *d*, is 0, *λ*/8, *λ*/4, and *λ*/2, respectively; **e** Purity of the generated BB while varying the antenna spacing.

This metric indicates the purity as 1 in the case of a perfect BB and 0 when the incident beam passes through RIS without attaining any phase. Figure 7a illustrates the transformation of a BB obtained in the presence of a vanishingly small element spacing or a continuous sheet—the ideal RIS. This is a perfect BB featuring an annular beam after the spatial Fourier transform. Figure 7b–d illustrates the Fourier transformation of the beam obtained when the antenna spacing, *d*, is *λ*/8, *λ*/4, and *λ*/2. Here we can notice a progressive fading of the annular beam intensity and a corresponding increase of the intensity at zero/low spatial frequencies. The above-defined purity of BB while varying the antenna elements’ spacing is plotted in Fig. 7e. We noticed a gradual decrease in the purity value with an increase in antenna spacing. Further details on the model are mentioned in the “Methods” section.

#### Impure Bessel beams’ ability to reconstruct

**Fig. 8:**
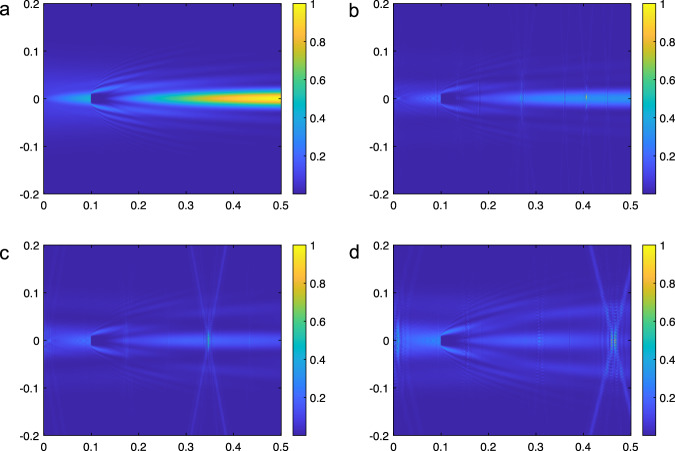
Impure Bessel beams (BBs) self-healing ability from 0 to 0.5 m. All the intensities are normalized (**a**) Perfect Bessel Beam (**b**) Bessel Beam with 75% Purity (*λ*/16 spacing (**c**) Bessel Beam with 50% Purity (*λ*/4 spacing) (**d**) Bessel Beam with 25% Purity (*λ*/2 spacing).

Now that we can quantitatively assess the quality of an RIS-generated BB, we proceed to verify whether they are able to self-heal after encountering an obstacle. Given that some of the BB’s properties are lost when using an RIS to generate it, it follows that the self-healing property may also be compromised. To explore this we use the same MATLAB simulation described above (and further in Methods) to simulate an RIS-generated BB and determine its purity level. We then propagate the BB through space where it encounters an obstacle. The obstacle is designed to block the central beam of the BB. The results are shown in Fig. 8a–d for Bessel beams with varying levels of purity.

As the purity of the BB diminishes, so does the strength of the reconstructed beam after the obstacle. Even so, with as low as 25% purity, which corresponds to antenna elements spaced a half-wavelength apart for 130 GHz, the beam still exhibits the ability to reconstruct in the presence of a partial blockage. These findings indicate that RIS-generated BBs are indeed a viable solution for mitigating the effects of blockage in THz near-field communications.

## Discussion

The results above demonstrate a notable advantage of utilizing THz BBs for communications in the near field, especially when mitigating blockage. Our analysis has been at sub-THz frequencies centered around 130 GHz, however the physics of sub-THz and true THz signals are the same, and thus our approach will scale to higher frequencies once the fabrication challenges of operating at true THz frequencies have been tackled. We have also included a detailed analysis on the self-healing nature of a BB by varying the topology of the obstacle/blockage. This analysis can be found in Supplementary Note 2.

From our experimental results we observe that Bessel beams operating within their focal range outperform Gaussian beams. BBs consistently outperform Gaussian beams in terms of SNR, EVM, and BER in the results at 20 cm. The improvement is maintained while increasing modulation order and/or the signal bandwidth. This observation is critical as high-data-rate communications often require high modulation orders and broad bandwidths. Moreover, when an obstacle is present the advantage of THz BBs is even more pronounced, allowing the receiver to observe signals that are completely imperceptible when a traditional Gaussian beam is used, and for THz communications, which rely heavily on strong line-of-sight paths, even partial blockage can greatly reduce the performance (as demonstrated in our experimental results with the Gaussian beam). Hence, choosing BBs over Gaussian beams for high-data-rate communications will be highly beneficial.

We proceed to define a method to quantitatively assess the quality of a BB and use simulations to demonstrate that RIS-generated BBs still retain their self-healing property. A major limitation of antenna arrays is the spatial phase discretization of each element, and we perform a systematic numerical analysis to illustrate its effects. Although conventional antenna arrays are usually designed with a spacing equal to *λ*/2 as this maximizes the beamforming gain without introducing grating lobes, metasurfaces can be designed with tightly packed and sub-wavelength-sized radiating elements. In this scenario, either more elements are required to preserve the directivity gain, which depends on the overall size of the structure, or the size between the elements must be increased. Thus an in important design question that follows is how the antenna element size and spacing impacts an RIS-generated BB’s ability to focus and to reconstruct after an obstacle. Our model acts as a tool to readily analyze this issue by allowing us to estimate the beam quality. As an example, we varied the spacing between the antenna elements to assess the quality of the generated beams and verified the beams’ ability to self-heal after partial blockage.

One key difference we observe between the perfect BB and the BBs with reduced levels of purity is that they focal line (which we can see form in around 35 cm in Fig. 8b) has reduced intensity for the RIS-generated BBs. Instead, the impure BBs contain a low intensity focal line accented periodically by focal points along the z-axis where the intensity is at its maximum. This discretization along the z-axis is a direct result of the discretization along the *y* and *x*-axes introduced by the spacing of the RIS elements. As the purity decreases, the intensity of the focal line diminishes and the focal points become more pronounced. Thus, even though BBs with low purity are still able to reconstruct, higher purity BBs would be preferable in order to closer mimic the focal line generated by a perfect BB.

In conclusion, we proposed BBs as an alternative to Gaussian beams for future high-data-rate near-field THz communications. To support this, we presented several experimental results acquired with THz BB generated with a 3D-printed axicon. The usage of BBs improves the characteristics of the THz communication link in the presence of an obstacle. We have also proven that this self-healing property is retained in RIS-generated BBs. As the next generation of communications shapes out, we believe that THz BBs can be a solid alternative to the traditional Gaussian beams at THz frequencies, due to their discussed advantages.

## Methods

### Fabrication

An axicon is a conical optical element typically used to transform Gaussian beams into Bessel beams. We fabricate an axicon using a commercial 3D printer (Ultimaker 3). The material used for the fabrication is Polylactic acid (PLA), with a refractive index of ~1.6 at 130 GHz frequency. The absorption losses of PLA are low in the sub-THz regime. In the 3D printer settings, we use an in-fill percentage of 100%, which results in an entire solid structure. The layer thickness and the speed of the printing process are optimized to yield a smooth finish.

### Simulation

We build a MATLAB model by defining the axicon phase and multiplying it with the Gaussian beam profile to mimic beam transformation occuring at the antenna face and 3D-printed axicon. The phase of axicon can be controlled in the model by varying the conical angle of axicon. After applying the pahse to the Gaussian Beam, we multiply the field with the RIS-antenna array system. The antenna array is an array of ones and zeros in a matrix; ones represent array elements of appropriate dimensions and zeros represent spacing between elements. The element dimensions are equal to the spacing of the elements. When multiply by a particular beam spatial phase, this antenna array will generate an equivalent discretized phase. We then use the Fresnel diffraction integral^47,48^ to estimate the beam profile as it propagates.

### Generation of info-bearing sub-THz modulated signals for experiments

In MATLAB, our data are organized into frames consist of a header for time synchronization, a 240 * *log*_2_(*M*)-bit pilot for post-equlization, followed by 12,000 * *log*_2_(*M*) random bits chosen for the data portion of the frame and no error correcting codes are used. The header is always modulated using BPSK, but the pilot and data portions are modulated using QAM at an intermediate frequency (IF) equal to half the bandwidth. This IF signal is pulse shaped to eliminate out-of-band transmissions. Next, the signal is passed from MATLAB to an Arbitrary Waveform Generator (AWG) where it is converted from a digital signal to an analog signal. This analog IF signal is then sent to the up-converter, which is powered by a DC power supply and a local oscillator (LO) to mix the IF signal to the desired radio frequency (RF) of 130 GHz. The LO we use generates a 32.5 GHz sinusoid that is passed through two frequency doublers to reach 130 GHz before being passed to the mixer.

On the receiver side, the reverse process is implemented by a down-converter before we use a digital storage oscilloscope to implement a low-pass filter (for noise mitigation) and to store a digitized version of the signal. This digitized signal is then loaded in MATLAB where we perform frame synchronization using the header, MMSE post-equalization using the pilot, and demodulation and detection of the data portion of the frame. More details on the experimental platform are given in Sen et al.^46^.

### Calculating experimental SNR, EVM, and BER values

Each performance metric is calculated directly from the received waveform. The SNR was calculated by computing the power of the data portion of the captured waveform and dividing it by the power observed during a silence we inserted into the frame for this purpose. The noise of our experimental system has a non-zero mean value. Thus to accurately calculate the power of the received signal and noise, we must subtract this mean value. Thus the power of the received signal and noise are given by

2 $${P}_{rx}=\frac{1}{{N}_{data}}{\Sigma }_{n = 1}^{{N}_{noise}}{x}_{data}{(n)}^{2}-\overline{{x}_{noise}},\,{{\mbox{and}}}$$

3 $${P}_{n}=\frac{1}{{N}_{noise}}{\Sigma }_{n = 1}^{{N}_{noise}}{x}_{noise}{(n)}^{2}-\overline{{x}_{noise}},$$

respectively. *N*_*data*_ and *N*_*noise*_ are the number of samples in the digitized data signal (*x*_*data*_) and noise signal (*x*_*noise*_). $\overline{{x}_{noise}}$ is the mean value of the noise. The EVM was calculated using the received IQ symbols according to *EVM* = *rms*(*IQ*_*tx*_ − *IQ*_*rx*_)/*rms*(*IQ*_*tx*_) * 100%.

### Supplementary information

Supplementary Material PDF

## Data Availability

Data supporting the findings of this study is available from the corresponding author on reasonable request in the form of MATLAB files.
