# Supplementary material for: Ultrabroadband terahertz-band communications with self-healing bessel beams
Source: Commun Eng. 2023 Oct 6;2:70. doi: 10.1038/s44172-023-00118-8 (PMC10955979; doi:10.1038/s44172-023-00118-8)
Supplement: Supplementary file 1 — Supplementary Material PDF [file 44172_2023_118_MOESM1_ESM.pdf]

Supplementary Information:
Ultrabroadband Terahertz-band Communications with Self-healing Bessel Beams

Innem V.A.K. Reddy[1,2], Duschia Bodet[3], Arjun Singh[4], Vitaly Petrov[3], Carlo Liberale[2,5], and Josep M. Jornet[3]
email:{j.jornet@northeastern.edu}
[1] Department of Electrical Engineering, University at Buffalo, SUNY Buffalo, Buffalo NY, USA,
[2] Biological and Environmental Science and Engineering Division, King Abdullah University of Science and Technology, Thuwal, Saudi Arabia,
[3]Department of Electrical and Computer Engineering, Institute for the Wireless Internet of Things, Northeastern University, Boston, MA, USA,
[4] Department of Engineering, SUNY Polytechnic Institute, Utica, NY, USA,
[5] Computer, Electrical and Mathematical Sciences and Engineering Division, King Abdulla University of Science and Technology, Thuwal, Saudi Arabia

Supplementary Note 1 - Transmitting images

To illustrate the high-data-rate communication scenario more closely, we also transmitted an image instead of a random stream of data bits. This way, errors would appear as distortions/noise in the received image at the Rx. The original source image is the publicly-accessible PNG file with the University logo (Supplementary Figure 1a). To avoid dependency on the file format, first, the image has been converted into a raw uncompressed bitmap of 200×200 pixels with a bit-depth of 1. When we first transmitted the image shown in Supplementary Figure 1a without any obstacle between the Tx and Rx, there were no errors in the received image (Supplementary Figure 1)b-e. The images exhibited minimal distortion / errors in both Gaussian and BB cases (64QAM and 256QAM). Next, we introduced an obstacle between the Tx and Rx and transmitted the same image (Supplementary Figure 1a) using either a Gaussian or a BB, while also changing the modulation (64QAM and 256QAM) order alongside. The received images are shown in Supplementary Figure 1f-i, and one can notice a significantly better quality received images while using a BB instead of a Gaussian beam.

Supplementary Note 2 - Varying the topology of the obstacle

Bessel beams are distinguished by their unique bullseye pattern that concentrates most of their power in the center. The subsequent rings carry the same amount of power as the central spot; however, the power is distributed radially. While blocking the beam, the most impact occurs if one blocks the central spot and the initial rings. For our experiment, we chose this scenario and used a cylindrical object. It blocks the center entirely along with a ring. The Bessel beam's rings aid in the healing process after an obstacle. However, it is equally interesting to investigate the obstacle topology dependence on the self-healing properties of a BB. Keeping this in mind, we conducted several studies by varying the size (both laterally and axially), rotation angle as well as the distance of the obstacle from the source.

Supplementary Figure 2 illustrates the impact of the width of the blocking object. Here, for convenience, we use relative width w.r.t. the physical size of the Tx antenna of 11.8 cm. In this figure, one can observe that: (i) the width of the obstacle plays a notable role in the link performance (particularly wider obstacle imposes a larger blockage zone behind it and

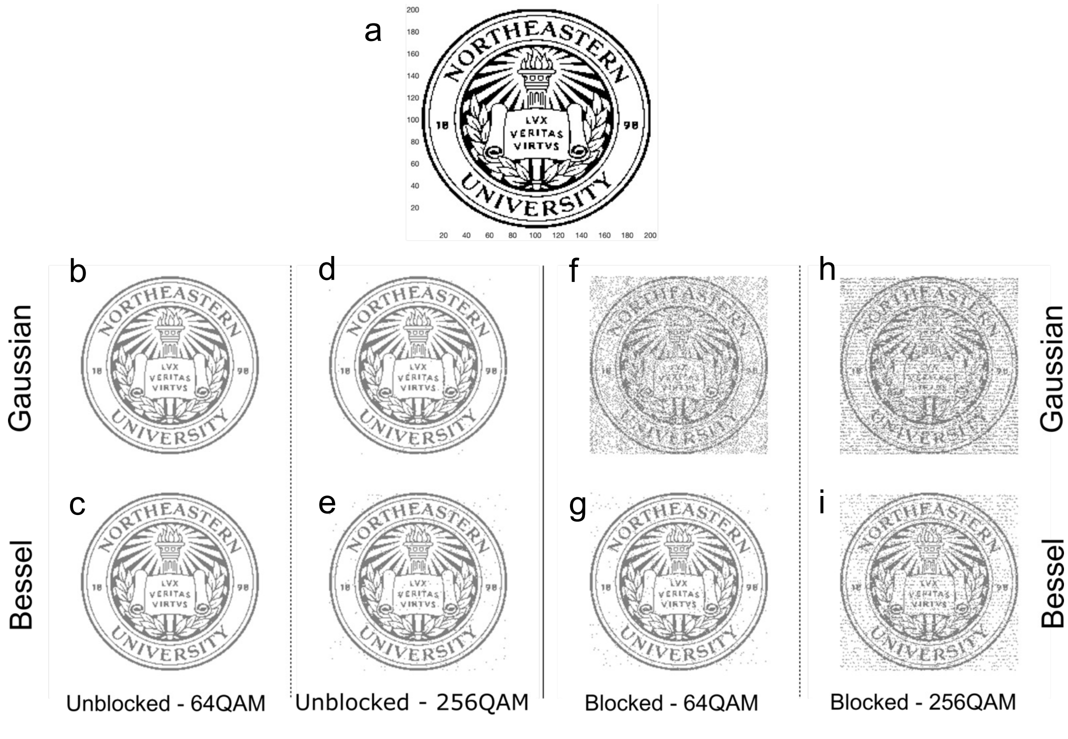

Supplementary Figure 1: Transmitted and received images; (a) Transmitted image with 200×200 pixels; (b-e) Received image with no blockage using Gaussian beam and Bessel beam (BB) – 64 Quadrature Amplitude Modulation (64 QAM) and 256 QAM modulation, (f-i) Received image with no blockage using Gaussian beam and BB – 64 QAM and 256 QAM modulation

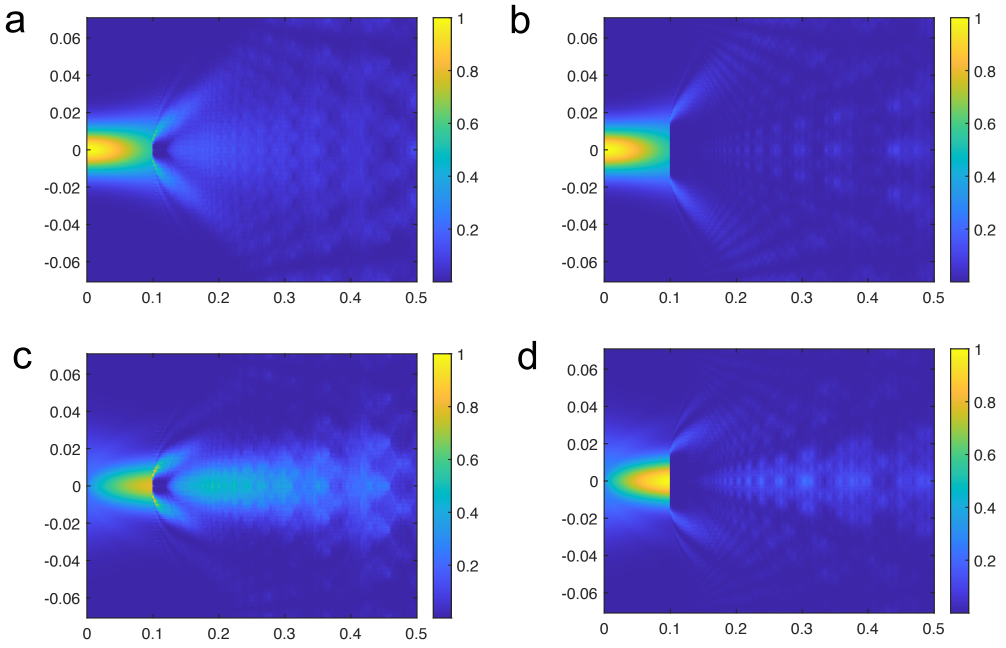

Supplementary Figure 2: Blockage impact and self-healing properties, different widths of the obstacle in relation to the transmitter (Tx) antenna size; (a) Gaussian beam with 20%-wide obstacle, (b) Gaussian beam with 80%-wide obstacle, (c) Bessel beam with 20%-wide obstacle, (d) Bessel beam with 80%-wide obstacle

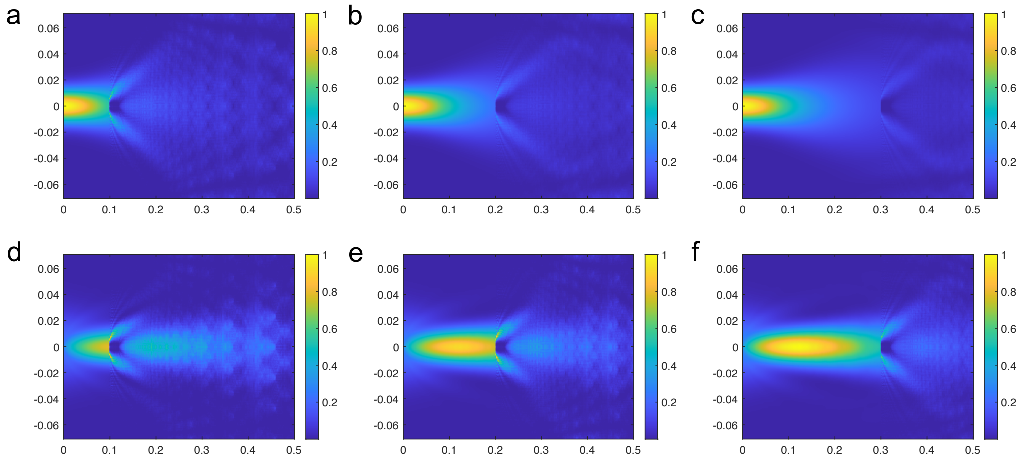

Supplementary Figure 3: Blockage impact and self-healing properties, different distances toward the blocker; (a) 10 cm distance, Gaussian beam, (b) 20 cm distance, Gaussian beam, (c) 30 cm distance, Gaussian beam, (d) 10 cm distance, Bessel beam, (e) 30 cm distance, Bessel beam, (f) 10 cm distance, Bessel beam

lower intensity/power at the receiver); (ii) (sub-)THz Bessel beam outperforms (sub-)THz Gaussian beam for a whole range of modeled obstacle sizes. However, it is important to note that the effects stay until very large relative widths of the blocker, where the entire beam becomes fully blocked (width $>$ 100% of the Tx antenna width). In the latter case, the full blockage is experienced and the performance of Gaussian and Bessel beams becomes comparable.

Supplementary Figure 3 illustrates the impact of the distance between the Tx antenna and the obstacle. Here, one can observe that the closer the obstacle is to the Rx (greater distance from the Tx) the worse the performance with the (sub-)THz Gaussian beam. The main reason for this effect is that the Rx stays deeper in the obstacle blockage zone, thus getting more shadowed by the obstacle body. In contrast, due to its specific properties, the (sub-)THz Bessel beam is less affected by the obstacle in different locations alongside the formed Bessel beam. In general, the obstacle just “cuts” a certain part of the Bessel beam, featuring a relatively small blockage (shadowing) zone and thus not impacting much the performance at the Rx. This scenario would only be problematic in the case that the obstacle is very close to the Rx, thus shadowing it from the transmitted (sub-)THz Bessel beam. Still, (sub-)THz Bessel beam outperforms (sub-)THz Gaussian beam in most of the modeled configurations.

We study the impact of the length of the obstacle itself in Supplementary Figure 4. From this figure, we observe that the impact of the obstacle length is: (i) less crucial compared to the obstacle width studied in Supplementary Figure 2 (discussed above) and (ii) intuitive, as longer obstacles “cut” larger parts of the path between the Tx and the Rx. However, the (sub-)THz Bessel beam can still be formed even after a 15 cm-long obstacle, thus demonstrating an advantage over the (sub-)THz Gaussian beam.

We finally proceed with Supplementary Figure 5 illustrating the impact of the obstacle rotation angle, when the overall size of the obstacle does not change notably. In this

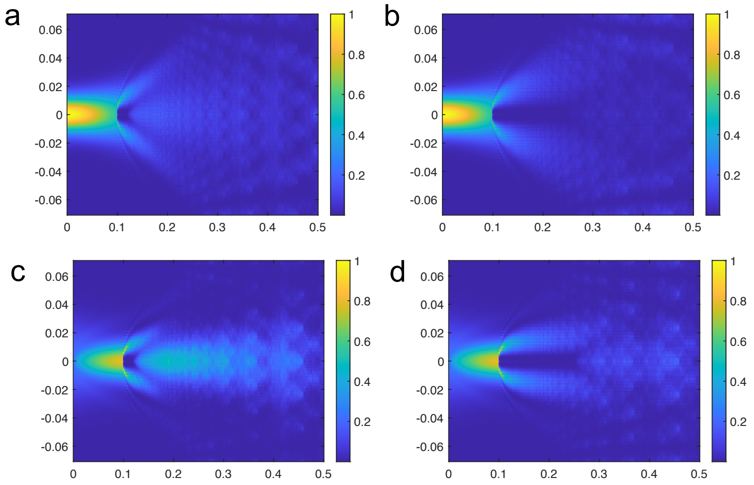

Supplementary Figure 4: Blockage impact and self-healing properties, different lengths of the obstacle; (a) 2 cm-long obstacle, Gaussian beam, (b) 15 cm-long obstacle, Gaussian beam, (c) 2 cm-long obstacle, Bessel beam, (d) 15 cm-long obstacle, Bessel beam

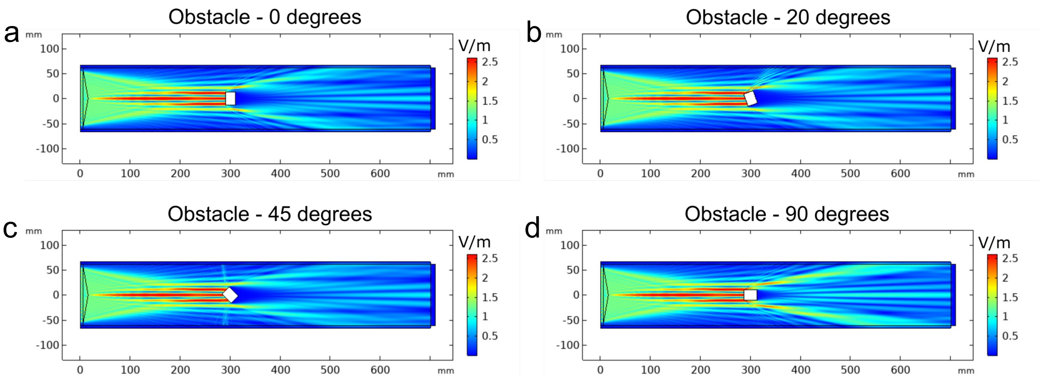

Supplementary Figure 5: Self-healing properties of Bessel Beams with rotated obstacles - (a) 0 degrees, (b) 20 degrees, (c) 45 degrees, and (d) 90 degrees

Supplementary figure, we compare different rotations of the similar-size obstacle. From this figure, one can notice minor changes in the self-healing property of the BB due to the obstacle rotations. Although the obstacle blocks the central spot along with the first ring, self-healing occurred due to the second and subsequent rings. Rotation of an obstacle did not interfere with the external rings, which is why we notice minor changes at the Rx end.

Summarizing this additional study, we conclude that, in the overwhelming majority of modeled configurations of partial blockage, the communication system utilizing the (sub-)THz Bessel beam outperforms the one exploiting the (sub-)THz Gaussian beam. This observation is made based on the extensive simulation campaign summarized above and also cross-checked for a subset of setups with real measurements using our test bed.

Figure Captions

Figure 1. Transmitted and received images; (a) Transmitted image with 200×200 pixels; (b-e) Received image with no blockage using Gaussian beam and Bessel beam (BB) – 64 Quadrature Amplitude Modulation (64 QAM) and 256 QAM modulation, (f-i) Received image with no blockage using Gaussian beam and BB – 64 QAM and 256 QAM modulation

Figure 2. Blockage impact and self-healing properties, different widths of the obstacle in relation to the transmitter (Tx) antenna size; (a) Gaussian beam with 20%-wide obstacle, (b) Gaussian beam with 80%-wide obstacle, (c) Bessel beam with 20%-wide obstacle, (d) Bessel beam with 80%-wide obstacle

Figure 3. Blockage impact and self-healing properties, different distances toward the blocker; (a) 10 cm distance, Gaussian beam, (b) 20 cm distance, Gaussian beam, (c) 30 cm distance, Gaussian beam, (d) 10 cm distance, Bessel beam, (e) 30 cm distance, Bessel beam, (f) 10 cm distance, Bessel beam

Figure 4. Blockage impact and self-healing properties, different lengths of the obstacle; (a) 2 cm-long obstacle, Gaussian beam, (b) 15 cm-long obstacle, Gaussian beam, (c) 2 cm-long obstacle, Bessel beam, (d) 15 cm-long obstacle, Bessel beam

Figure 5. Self-healing properties of Bessel Beams with rotated obstacles - (a) 0 degrees, (b) 20 degrees, (c) 45 degrees, and (d) 90 degrees
